# Biotic stress in plants: life lessons from your parents and grandparents

**DOI:** 10.3389/fgene.2012.00256

**Published:** 2012-12-06

**Authors:** A. E. Dorantes-Acosta, C. V. Sánchez-Hernández, M. A. Arteaga-Vázquez

**Affiliations:** ^1^Laboratorio de Epigenética y Biología del Desarrollo, Instituto de Biotecnología y Ecología Aplicada(INBIOTECA), Universidad Veracruzana.Xalapa, Veracruz. México; ^2^Departamento de Producción Agrícola, Centro Universitario de Ciencias Biológicas y Agropecuarias, Universidad de GuadalajaraGuadalajara, Jalisco, Mexico

Epigenetic regulation is essential for growth and development in eukaryotic organisms (Henikoff et al., 2008; Suzuki and Bird, 2008) and is also responsible for the establishment, maintenance, and reversal of non-genetic cellular memory that records developmental and environmental cues, including those arising from biotic and abiotic stress (Bonasio et al., 2010). A series of recent stimulating papers, show that biotic stress can trigger a transgenerational epigenetic response in plants, where DNA methylation seems to play a central role.

Plants sense and respond to environmental cues by a repertoire of mechanisms that regulate gene expression in order to maximize chances of survival in hostile environments. In addition to preformed defense traits, plants have evolved inducible defenses against microbial pathogens, herbivores, and even other plants that involve the regulation of gene expression for the synthesis of defensive secondary metabolites and specific proteins (Walling, 2000; Howe and Jander, 2008; Mithofer and Boland, 2012). Plants rely on the innate immunity of each cell and on systemic signals emanating from infection sites (Jones and Dangl, 2006). Plant hormones play essential roles during systemic defense signaling (Robert-Seilaniantz et al., 2011). Salicylic acid (SA) primarily triggers resistance against biotrophic and hemibiotrophic pathogens whereas a combination of jasmonic acid (JA) and ethylene (ET) signaling activates resistance against necrotrophic pathogens (Glazebrook, 2005; Robert-Seilaniantz et al., 2011). SA acts as an endogenous signal involved in systemic acquired resistance (SAR), an inducible resistance against a broad spectrum of pathogens including viruses, bacteria, and fungi that cause necrosis through rapid programmed cell death of infected cells, known as the hypersensitive response (Durrant and Dong, 2004). SAR induces resistance in the systemic (uninoculated) plant organs in response to local infection (Vlot et al., 2009). Recent evidence shows that NON-EXPRESSOR of PATHOGENESIS-RELATED GENES 3 (NPR3) and NPR4 are SA receptors that bind SA with different affinities and regulate degradation of the transcription cofactor NPR1 in a SA-dependent manner (Fu et al., 2012).

In addition to their role during plant development, JA and JA-related compounds, including methyl-jasmonate (MeJA) and jasmonate-isoleucine conjugate (JA-Ile), play essential roles during endogenous regulation of plant resistance to mechanical wounding and herbivory by modulating global changes in gene expression (Creelman and Mullet, 1997; Sheard et al., 2010). Priming (or sensitization) refers to the enhanced ability for the quicker and more effective activation of specific cellular defense responses upon previous exposure to biotic or abiotic stress (Conrath et al., 2002, 2006). Defense pathways and priming can also be induced by application of chemical stimuli including the non-protein amino acid beta-amino-butyric acid (BABA) (Zimmerli et al., 2000). In addition to plant hormones, small RNAs and genes involved in the biogenesis of small RNAs are also components of the plant defense strategies against herbivory and microbial pathogens (Pandey et al., 2008; Ruiz-Ferrer and Voinnet, 2009; Katiyar-Agarwal and Jin, 2010).

Here we comment on a series of papers that provide evidence on transgenerational epigenetic effects during biotic stress. Rasmann et al. (2012) report transgenerational priming responses (TPR) in tomato and Arabidopsis induced by caterpillar herbivory (*Helicoverpa zea* and *Pieris rapae*) and application of MeJA. TPR results in a reduction of up to ~40% in caterpillar weight gain and this effect persists after one stress free generation in Arabidopsis (Rasmann et al., 2012). Experiments with the *coronatine insentitive1* (*coi1-1*) and the triple *dicer-like2/dicer-like3/dicer-like4* (*dcl2/dcl3/dcl4*) mutants show that the TPR depends on JA perception and on components of the RNA-dependent DNA methylation pathway involved in *de novo* DNA methylation (RdDM) (Law and Jacobsen, 2010; Rasmann et al., 2012).

In order to assay transgenerational effects in response to the biotrophic pathogen *Pseudomonas syringae* pv *tomato* DC3000 (*Pst*DC3000) in Arabidopsis, Luna et al. (2012) recurrently inoculated a set of parental lines (P0) with increasing doses of *Pst*DC3000. Fitness of progeny (P1) from *Pst*DC3000 infected plants did not differ statistically from control plants progeny (C1) (Luna et al., 2012). However, P1 plants showed a statistically significant reduction in the colonization and disease symptoms after inoculation with: (1) the oomycete pathogen *Hyaloperonospora arabidopsidis* and (2) *Pst*DC3000-lux (a bioluminescent strain of *Pst*DC3000). Once established, this TPR can be maintained over one stress-free generation. Similar experiments employing parental lines in an *npr1-1* mutant background showed that colonization reduction by *H. arabidopsidis* depends on a functional *NPR1* gene. Consistent with an NPR1-dependent priming of SA-inducible defense, a faster and stronger induction of SA-inducible defense genes such as *PATHOGENESIS-RELATED GENE 1* (*PR1*), *WRKY6*, *WRKY53*, and *WRKY70* was also observed in P1 plants (Luna et al., 2012). Antagonistic effects between JA and SA pathways were confirmed by inoculating P1 plants with the nectrotrophic fungus *Alternaria brassicicola* (an inducer of JA-dependent defense response). P1 plants contained similar endogenous levels of JA, JA-Ile, JA-precursor, and SA to C1 plants, but showed increased hyphal colonization and a reduction in the expression of JA-inducible genes, including *PLANT DEFENSINE 1.2* (*PDF1.2*). The priming effects on SA and JA inducible genes correlate with the NPR1-dependent deposition of epigenetic marks characteristic of active chromatin (H3K9ac; acetylation of Lys-9 on Histone 3) in the case of *PR-1*, *WRKY6* and *WRKY53*; and repressive chromatin (H3K27me3; trimethylation of Lys-27 on Histone 3) in the case of *PDF1.2*. Although a role for chromatin modifications mediating the TPR wasn't ruled out, assays with the *domains rearranged methyltransferase 1 and 2/cytosine methyltransferase 3* (*drm1/drm2/cmt3)* triple mutant, affected in DNA methylation, showed that this mutants mimics the TPR observed in P1 plants and indicates that components of the RdDM pathway are required for TPR (Luna et al., 2012).

Similar experiments from Slaughter et al. (2012) using either BABA or an avirulent *Pst* strain also showed a TPR against *Pst*DC3000 and *H. arabidopsidis*, and an *IBS1* (*IMPAIRED BABA-INDUCED STERILITY RESPONSE 1*)-dependent increased expression of *PR1*, *PR2*, and *PR5*. However, in sharp contrast to Luna et al. and Rasmann et al., the TPR was only maintained in the immediate progeny which might be the result of differences in experimental approaches (recurrent vs. single inoculation/stimulation) or actually represent biological differences in the molecular mechanism(s) involved (Slaughter et al., 2012).

High resolution genomewide profiling of the DNA methylation landscape in Arabidopsis from Dowen et al. (2012) shows that global disruption of establishment and maintenance of DNA methylation in a set of mutants including *drm1/drm2/cmt3* and *methyltransferase 1* (*met1*) enhances resistance to bacteria and induces widespread dynamic changes in methylation. Distinct patterns of differentially methylated regions (DMR) can be observed when wild type plants are exposed to SA or either virulent or avirulent *Pst* strains. SA treatment uncovered a class of 21-nt siRNAs particularly evident at transposable elements (TEs)–associated DMRs and in many cases, SA-induced DMR associate with reprogramming of TEs and neighboring genes (Dowen et al., 2012).

Taken together, this set of papers unveiled a TPR during biotic stress in plants where DNA methylation and components of the RdDM pathway play a major role. Some open questions: what is the molecular nature of the TPR signal? What cells are responsible for sensing and transmitting the biotic stress memory? What is the impact of plant's age and intensity of the biotic stress on the TPR? Can the TPR behave as a paramutation-like phenomena?
